# Treatment of pediatric lateral condylar humerus fractures with closed reduction and percutaneous pinning

**DOI:** 10.1186/s12891-020-03738-9

**Published:** 2020-10-27

**Authors:** Li-wei Xie, Juan Wang, Zhi-qiang Deng, Ren-huan Zhao, Wei Chen, Chi Kang, Jia-jun Ye, Xin Liu, Ying Zhou, Hai Shen

**Affiliations:** 1 Department of Pediatric Orthopedics, Sichuan Provincial Orthopedics Hospital, Chengdu, Sichuan China; 2 Department of Geriatrics, Chengdu Shuang-nan Hospital, Chengdu, Sichuan China

**Keywords:** Lateral condylar humerus fractures, Children, Closed reduction and percutaneous pinning, Arthrogram

## Abstract

**Background:**

Lateral condylar humerus fractures (LCHFs) are the second most common pediatric distal humerus fractures. Open reduction and internal fixation is recommended for fractures displaced by more than 2 mm. Few studies described using closed reduction and percutaneous pinning (CRPP) for treating fractures with greater displacements. This study aims to explore the feasibility of CRPP in treating displaced LCHFs.

**Methods:**

All patients underwent attempted CRPP first. Once a satisfying reduction was obtained, as determined using fluoroscopy based on the relative anatomical position of the fragments, an intraoperative arthrogram was performed to further confirm the congruence of the articular surface of the distal humerus. Open reduction is necessary to ensure adequate reduction if the fracture gap is more than 2.0 mm on either anteroposterior view or oblique internal rotational view by fluoroscopy after CRPP. All included fractures were treated by a single pediatric surgeon.

**Results:**

Forty-six patients were included, 29 boys and 17 girls, with an average age of 5.2 years. Of these, 22/28 (78%) Jakob type II fractures and 14/18 (78%) Jakob type III fractures were treated with CRPP. All cases in Song stages II and III, 19/25 (76%) cases in Song stage IV, and 14/18 (78%) cases of Song stage V were treated with CRPP. The remaining converted to open reduction with internal fixation. Overall, 36 of the 46 patients (78%) were treated with CRPP. The average pre-op displacement was 7.2 mm, and the average post-op displacement was 1.1 mm on the anteroposterior or oblique internal rotational radiograph in cases treated with CRPP. CRPP was performed in an average of 37 min. The average casting period was 4 weeks and the average time of pin removal was 6 weeks postoperatively. The average time of follow-up was 4 months. All patients achieved union, regardless of closed or open reduction. No infection, delayed union, cubitus varus or valgus, osteonecrosis of the trochlea or capitellum, or pain were recorded during follow-up.

**Conclusions:**

Closed reduction and percutaneous pinning effectively treats LCHFs with displacement more than 4 mm. More than 3/4 of Song stage V or Jakob type III patients can avoid an incision.

## Background

Lateral condylar humerus fractures are the second most common distal humeral fracture in children, accounting for approximately 17% of pediatric distal humerus fractures, occurring at an average age of 6 years old [1]. The fractures are usually unstable and tend to displace as a result of activities of the hands and wrists because the lateral condyle is the origin of common extensor, which may lead to nonunion and cubitus valgus deformity of the elbow joint if treated improperly [2]. These fractures are usually intra-articular [1]. Traditionally, open reduction with percutaneous pinning has been preferred by majority of surgeons to insure anatomic reduction of these physeal, intra-articular fractures [3]. Furthermore, several reports have recommended open reduction and internal fixation (ORIF) as the best intervention for unstable fractures to prevent further displacement, nonunion, and malunion [4–8]. However, closed reduction and percutaneous pinning (CRPP) for treating pediatric lateral condylar humerus has been reported only in a few studies [9–18]. CRPP has shown several advantages over ORIF, including less dissection of soft tissue around the fragment, low risk of vessel damage, low risk of non-union and avascular necrosis (AVN) of distal humerus physeal, shorter operating room times, and avoidance of an open incision with an unaesthetic scar [15–19]. In most studies, this technique was used only in cases with displacement between 2 and 4 mm [9, 12, 15]. Song et al. introduced a new classification and an internal oblique view to better assess the displacement of the fragment [20], and they managed to achieve closed reduction of the fracture, even in cases with displacement exceeding 4 mm or those with rotational displacement [13, 14]. They also pointed out that this technique has a difficult learning curve [1, 13, 14]. This study aims to explore the efficacy and safety of this technique in a retrospective way.

## Methods

### Patients

After obtaining informed consent from the guardians and approval of our institutional review board, we retrospectively reviewed 46 consecutive displaced lateral condylar humerus fractures from June 2019 to April 2020 at our institution. All patients were treated by a single senior attending pediatric orthopedic surgeon in our pediatric orthopedic department. The inclusion criteria were as follows: age below 14 years; closed fractures; fresh lateral condylar humerus fractures; displacement of fragments exceeding 2 mm; or unstable fractures with less than 2 mm displacement. The exclusion criteria were as follows: age above 14 years; open fractures; old fractures; and stable fractures or fractures with displacement less than 2 mm. Both the well-known Jakob classification and a novel but more detailed Song classification were used to classify the fractures [12, 20]. (Table 1).

**Table 1:** The Song classification of pediatric lateral condylar humerus fractures

Stage	Degree of displacement	Fracture pattern	Stability
I	≤2 mm	Limited fracture line within the metaphysis	Stable
II	≤2 mm	Fracture line extends to the epiphyseal articular cartilage with a Lateral gap	Indefinable
III	≤2 mm	Gap as wide laterally as medially	Unstable
IV	>2 mm	Without rotation of fragment	Unstable
V	>2 mm	With rotation of fragment	Unstable

Modified from: Song KS, Kang CH, Min BW, et al. Closed reduction and internal fixation of displaced unstable lateral condylar fractures of the humerus in children. J Bone Joint Surg Am. 2008; 90(12):2673–2681

### Surgical technique

**Fig. 1:**
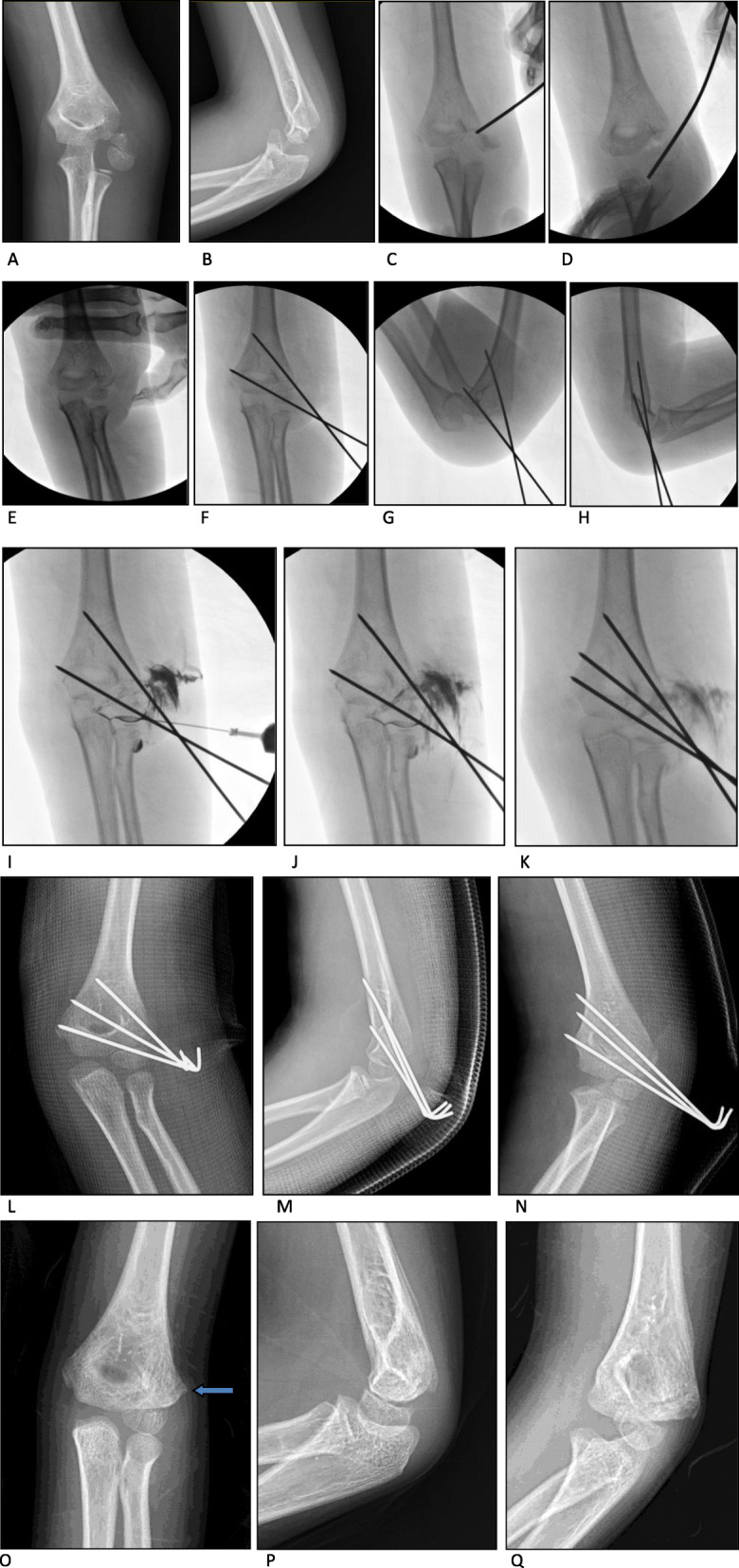
A Jakob type III and Song stage V fracture treated with CRPP. **a**-**b**: pre-operative films of the fracture. **c**-**e**: closed reduction of the fracture with a 2.0-mm Kirschner wire as a joystick and manipulation. **f**-**h**: percutaneous pinning of the fracture after reduction. **i-k**: intraoperative arthrogram through a lateral puncture point showed congruence of the articular surface. **l-n**: post-operative anteroposterior view, lateral view and oblique internal rotational films of the fracture showed nearly anatomic reduction of the fracture. **o-q**: X-rays showed union of the fracture after pins’ removal, the arrow showed the bone spur formation on the lateral side of distal humerus

The patients lay on the operating table in a supine position with the affected arm abducted. After induction of general anesthesia, a tourniquet was applied in case of opening reduction followed by skin preparation and draping. To reduce the fracture in closed fashion, a gentle traction with the forearm in supination and a varus force was applied to the affected elbow joint to create a space for the reduction of the fragment. Then, a gradual compression on the distal fragments both medially and anteriorly by the surgeon’s thumb was applied, followed by a valgus force and extension of the elbow to reduce the lateral and posterior gaps of fragments and maintain the reduction. This manipulation can help achieve a fracture reduction in most cases with distal fragment without complete rotation (Song stage V and Jakob type III). However, in cases with rotated distal fragments, an anterior backward and inward compression of the distal fragment with the elbow joint flexion over 90° corrects the anterolateral and rotational displacement; otherwise, a Kirschner wire with a diameter of 2.0 mm was inserted percutaneously as a joystick into the space between the fragments. Then, a direct backward and inward force was applied to the fracture surface of the distal fragment with the elbow in flexion and medial compression by the surgeon’s thumb. Usually, the rotation can be corrected by such a maneuver; if not, another attempt may be needed. Then, reduction of the fracture was performed using a method similar to that followed for fracture fixation without fragment rotation. Once a satisfying reduction was achieved with the relative anatomical position of the fragments confirmed using fluoroscopy, percutaneous pin fixation with three lateral divergent 1.5-mm Kirschner wires was performed to stabilize the fragments. Then, an intraoperative arthrogram was used to confirm the congruence of the articular surface of the distal humerus. However, ORIF is necessary to ensure adequate reduction if the fracture gap is more than 2.0 mm on either the anteroposterior view or oblique internal rotational view in fluoroscopy after CRPP. Postoperatively, the arm was immobilized at 70° of elbow flexion with a posterior long-arm cast, which was planned to be removed 4 weeks postoperatively. The pins were removed 1 week later depending on the extent of union of fractures. All patients were followed up and complications were recorded. Function of the elbow joint was graded according to the criteria of Hardacre [21]. (Fig. 1).

## Results

In total, 46 patients were included in the study at our center during the study period. Of these, the fracture type was Jakob type II in 28, Jakob type III in 18, Song classification stage II in 1, stage III in 2, stage IV in 25, and stage V in 18. There were 29 boys and 17 girls, with an average age of 5.2 years (range: 1.8–9.7 years). All fractures were treated by a single senior attending pediatric orthopedic surgeon. All cases underwent attempted closed reduction and percutaneous pinning first. If a fracture gap less than 2 mm could not be gained, an open reduction and internal fixation would be performed. Among the 46 patients, 22/28 cases (78%) with Jakob type II and 14/18 cases (78%) with Jakob type III fractures were treated with CRPP. All fracture cases of Song stages II and III, 19/25 (76%) cases of Song stage IV, and 14/18 (78%) cases of Song stage V were treated with CRPP. The remaining were transferred to ORIF. In all, 36 of 46 (78%) were treated with CRPP. The average preoperative displacement was 7.2 mm (range: 1.5–20 mm) on the anteroposterior or oblique internal rotational radiograph. The average postoperative displacement was 1.1 mm (range: 0.5–1.8 mm) on the anteroposterior or oblique internal rotational radiograph in cases treated with CRPP. The average preoperative displacement was 11.8 mm (range: 7–20 mm) in Song stage V and 5.0 mm (range: 2.1–9 mm) in Song stage IV LCHFs, and the average postoperative displacement was 1.2 mm (range: 0.3–2 mm) in Song stage V and 1.1 mm (range: 0.5–2 mm) in Song stage IV LCHFs. The average operation time was 47 min, with an average of 37 and 77 min for CRPP and transferred ORIF. The average length of hospital stay was 5 days (range: 3–7 days). The average casting period was 4 weeks (range: 3–6 weeks), and the average time of pin removal was 6 weeks (range: 4–8 weeks) postoperatively. The average time of follow up was 4 months (range: 3–6 months). All cases achieved union, regardless of whether they were treated with closed or open reduction. All cases achieved satisfied range of motion except 3 cases, but the result was good according to Hardacre’s criteria. No infection, nonunion, cubitus varus or valgus, osteonecrosis of the trochlea or capitellum, or pain were recorded during follow-up. Lateral condylar bone spur formation was found in 89% (41/46) of all cases without any symptom. In cases converted to ORIF, the incidence of bone spur deformity was 100% (10/10), while in cases treated with CRPP, the incidence of bone spur deformity was 86% (31/36). According to the criteria of Hardacre et al., the clinical result was excellent in 33/36 (92%) patients, good in 3/36 (8%) patients (2 was in the ORIF cases, 1 in the CRPP cases), and poor in no patients undergoing CRPP.

## Discussion

Lateral condylar humerus fractures are the second most common distal humerus fractures in children, accounting for approximately 17% of pediatric distal humerus fractures [1]. Treatment of the fractures depends on the extent of displacement of the distal fragment and stability of the fracture. Usually, for nondisplaced and minimally (less than 2 mm) displaced stable fractures, posterior plaster splint or long-arm cast followed by close observation usually produces good results; while for those displaced unstable fractures, open reduction and internal fixation is usually recommended to obtain a congruent articular surface and sufficient fracture reduction. This treatment strategy has been widely accepted by majority of pediatric surgeons [1–8].

Few studies reported on CRPP for treatment of pediatric lateral condylar humerus fractures. Foster et al. reported that percutaneous pin fixation of nondisplaced and minimally displaced (<2 mm) fractures is an acceptable alternative in any situation in which close scrutiny cannot be ensured [9]. Mintzer et al. treated 12 children with lateral condyle fractures with moderate displacement (2–4 mm) using CRPP. The intraoperative arthrograms showed no incongruity of the articular surface and the results were good without complications. They advocated CRPP for unstable, moderately displaced lateral condylar fractures (Jakob type II) [10]. Weiss et al. reported 65 patients with lateral condyle fractures displaced > 2 mm but with an intact articular surface as assessed by an arthrogram, treated using CRPP. They claimed that fractures with displacement more than 4 mm were more likely to have incongruent articular surface, which should be treated with open reduction [11]. However, all these cases treated with CRPP were fractures with minimal or moderate displacement (<4 mm) [9–11]. It appears that it is impossible to treat such fractures as Jakob type III using CRPP.

Song et al. showed that significant displaced unstable lateral condylar humerus fractures, even those with totally rotation, could be managed without open reduction [12–14]; at this point, CRPP truly become a viable option. They attempted CRPP in all cases; 13 (76%) of 17 stage III fractures, 30 (75%) of the 40 stage IV fractures, 3 (50%) of the 6 stage V fractures were successfully treated by CRPP with residual displacement less than 2 mm. The follow-up showed excellent or good results in all cases with minor complications like bone spurs at the lateral side of the distal humerus without symptoms. Their later papers, including more fractures with completely and rotational displacement, showed higher success rates. Eighteen of 24 (75%) completely displaced and rotated fractures were reduced within 2 mm of residual displacement using closed reduction and internal fixation (CRIF). Therefore, they claimed that CRIF is an effective treatment for completely displaced and rotated LCHFs in many children [12–14].

Our study showed similar results in treating LCHFs with displacement more than 4 mm by CRPP. Approximately 78% (36/46) of our series were managed with CRPP, with success rate of 76% (19/25) for Song stage IV and 78% (14/18) for Song stage V fractures, and 78% (22/28) in Jakob type II and 78% (14/18) in Jakob type III fractures. The final follow-up showed no functional disfunction or complications except lateral bone spur formation without symptoms in most cases.

Now this CRPP technique has been introduced in the latest edition of a classic book, Rockwood and Wilkins Fractures in Children [1]. A few later studies reported their results using this technique [15–18]. Silva et al. claimed that CRPP is an alternative treatment of pediatric LCFs with displacement between 2 and 4 mm. The advantages of this technique include avoiding an unaesthetic scar, decreased surgical times, and not significantly increasing the incidence of complications [15]. The study by Pennock et al. reported that only one patient with displacement between 4 and 5 mm in their series was treated with CRPP; the authors concluded that CRPP may be suitable for lateral condyle fractures with displacement between 2 and 4 mm, because of high incidence of joint congruity in these cases, thereby avoiding an incision and decreasing surgical time [16].

However, almost all cases, in these studies were LCHFs with displacement less than 4 mm. The reason may be the difficult learning curve, as pointed out by Song et al. [12]. In the present study, the average preoperative displacement was 11.8 mm in Song stage V and 5.0 mm in Song stage IV LCHFs, with an average postoperative displacement was 1.2 mm in Song stage V and 1.1 mm in Song stage IV LCHFs. All cases were treated by a single surgeon (Li-Wei Xie) in our department, who had little experience in treating LCHFs with CRPP, but abundant experience with ORIF and in treating supracondylar fractures with CRPP. The authors’ experiences in this study showed that CRPP in treating LCHFs is technically feasible, and that more than 3/4 of cases can avoid an incision. The surgeon should prepare himself to make sure he is familiar with the technique of manual reduction before starting his first case.

The following are several useful tips to achieve a satisfied reduction of the fractures. First, never do an arthrogram before reduction, as this will interfere with your judgement regarding the relative position of the fragments. Because the origin of the extensor of the forearm is always disrupted, contrast medium will flow into the lateral soft tissue and this will blur the pictures of lateral side of the distal fragment. Second, a lateral puncture point for arthrogram could be used. The position is just at the lateral side of the humeroradial joint. In this way, contrast medium will directly flow into the articular surface and the fracture gap with little disturbance of judgement of the reduction. Third, the distal fragment will displace anteriorly or posteriorly on the lateral fluoroscopy after reduction, which could be reduced by extension or flexion of the elbow joint. Fourth, the pinning site is a little more distal and anterior than the regular position in pinning a flexed elbow, because this manipulation usually needs to maintain the reduction in an extensional elbow with a valgus force. Fifth, for Jakob type III and Song stage V, the difficulty lies in the correction of rotation of the distal fragment. In most cases, an anterior backward and inward compression of the distal fragment with the elbow joint flexion could correct the rotational displacement in most cases. Occasionally, a 2.0-mm Kirschner wire could be used as a joystick to help reduce the rotation. Sixth, a thinner 1.0-mm Kirschner wire could be used first to avoid too much pinning holes in the small nuclear ossification of distal physeal, which could be changed to 1.5-mm Kirschner wires for final fixation. Last but not least, if the reduction of a LCHF cannot be obtained by several attempts, convert to ORIF directly.

The present study has several limitations. The number of cases was small and the time of follow-up was short. Because the study was not controlled, we could not tell the difference between cases treated with CRPP and the cases treated with ORIF directly. Moreover, the cases in this study were performed by one surgeon, and we are not sure about the repeatability among surgeons.

## Conclusions

CRPP has shown several advantages over ORIF, including less dissection of soft tissue around the fragment, low risk of vessel damage, low risk of non-union and AVN of distal humerus physes, shorter operating room times, and avoidance of an open incision with unaesthetic scar [15, 19], avoidance of another operation or anesthesia because of hardware removal. The present study demonstrated that closed reduction and percutaneous pinning is a safe and efficient technique for treating LCHFs with displacement more than 4 mm, and more than 3/4 of Song stage V or Jakob type III patients can avoid an incision. Post-fixation intro-operative arthrography is a useful method for accessing the congruence of articular surface and the displacement of the fragment.

## Data Availability

The data and materials in the current study are available from the corresponding author on reasonable request.

## Abbreviations

LCHFs: Lateral condylar humerus fractures
CRPP: Closed reduction and percutaneous pinning
ORIF: Open reduction and internal fixation
AVN: Avascular necrosis
